# Limits on performance and survival of juvenile sockeye salmon (*Oncorhynchus nerka*) during food deprivation: a laboratory-based study

**DOI:** 10.1093/conphys/coab014

**Published:** 2021-03-24

**Authors:** Samantha M Wilson, Kendra A Robinson, Sarah Gutzmann, Jonathan W Moore, David A Patterson

**Affiliations:** 1 Earth to Ocean Research Group, Simon Fraser University, 8888 University Drive, Burnaby, British Columbia V5A 1S6, Canada; 2 Fisheries and Oceans Canada, Cooperative Resource Management Institute, School of Resource and Environmental Management, Simon Fraser University, Burnaby, British Columbia V5A 1S6, Canada

**Keywords:** Carryover effects, condition factor, energy density, migration, sockeye salmon, survival, swim performance

## Abstract

Long-distance migrations can be energetically demanding and can represent phases of high mortality. Understanding relationships between body condition and migratory performance can help illuminate the challenges and vulnerabilities of migratory species. Juvenile anadromous sockeye salmon (*Oncorhynchus nerka*) may migrate over 1000 km from their freshwater nursery habitats to estuary and ocean feeding grounds. During the period corresponding to the seaward migration of sockeye salmon, we held smolts in the laboratory to ask the following: (i) Does non-feeding migration duration influence prolonged swim performance and survival? (ii) What are the relationships between individual body condition and swim performance and survival? Wild sockeye salmon were intercepted during their migration and held without food for up to 61 days to represent the non-feeding freshwater migration and the extremes of poor estuary habitat. We conducted 40 sets of prolonged swim trials on 319 fish from 3 treatment groups that represented entrance to the marine environment on (i) an average,(ii) a delayed and (iii) a severely delayed migration schedule. Experimentally controlled freshwater migration duration did not impact swim performance or survival. Swim performance decreased concomitant with condition factor, where smolts with a Fulton’s condition factor of <0.69 were less likely (<50% probability) to complete the swim test (90 min swim test, at ~0.50 m/s). Survival of salmon smolts in the laboratory was less likely at energy densities of less than 3.47 MJ/kg. Swim performance decreased much sooner than survival, suggesting that swim performance, and therefore condition factor, may be a good indicator of survival of migratory smolts, as fish with reduced swim performance will likely be predated. These two relationships, one more ecologically relevant and one more clinical, help reveal the limits of long-distance migration for juvenile salmon and can be used to determine population-specific starvation risk associated with various freshwater and marine habitat conditions.

## Teaser

Sockeye salmon smolts can undergo vast migrations without feeding. We explored relationships between deteriorating body condition and swim performance/survival. Most fish could not complete a swim challenge after 3 weeks without food, and after ~8 weeks, 50% had perished. Condition factor and energy density predicted swim performance and survival, respectively.

## Introduction

Long-distance migrations are often challenging life history phases with higher mortality rates compared to stationary phases (Sillett and Holmes, 2002; Alerstam *et al.*, 2003; Klaassen *et al.*, 2014; Lok *et al.*, 2015; Clark *et al.*, 2016). Migratory success or failure may be controlled by variation in individual condition and energetics within and across populations. For instance, body condition can be a strong indicator of migration success (Drent *et al.*, 2003; Duijns *et al.*, 2017), as long-distance migrants often rely heavily on endogenous energy stores to fuel migrations (McKeown, 1984; Dingle, 1996) and could be a potential tool for predicting migration success. For example, red knots (*Calidris canutus rufa*) with higher body condition (size-corrected mass) had faster migration to breeding grounds, higher migration success (survival), and were more likely to have bred successfully, than those of lower body condition (Duijns *et al.*, 2017). Lower quality individuals are also more likely to be predated during migration (Dierschke, 2003; Tucker *et al.*, 2016). Thus, body condition at the beginning of the migration can influence subsequent performance, thereby driving carryover effects across life stages and habitats. Understanding the relationship between body condition and the survival of migrating animals could help clarify their limits and vulnerabilities to environmental or anthropogenic disturbances in habitat or migratory conditions.

While energy and migration success are presumably related for many populations (Drent *et al*., 2003), these relationships remain relatively poorly described for many important migratory species. It is possible that there is an abrupt threshold, where performance remains robust until physical or energetic condition decreases to a threshold at which point performance decreases dramatically. Alternatively, the relationship could be more linear, where a unit decrease in condition would result in a proportional decrease in performance (Huggett, 2005; Ficetola and Denoël, 2009). It is also possible that there is not a single condition metric that accurately predicts performance given the number of different physiological processes that support a single type of performance. Understanding the form of condition–performance relationships, and the thresholds they may contain, is important for predictions of migration success. For example, identifying an energetic condition–performance threshold could help identify the proportion of individuals in a population that may be at risk of not completing their migration. Such thresholds could also be used to understand the vulnerabilities of different populations to changes in migratory conditions, such as anthropogenic flow alteration that results in slowed migration rates (Raymond, 1968, 1979).

Juvenile Pacific salmon (*Oncorhynchus* spp.) complete long-distance, and sometimes energetically expensive, migrations from their natal rearing lakes and streams to estuarine and ocean feeding grounds (Brett, 1995; Hinch *et al.*, 2006). These freshwater migrations range in distance from tens to over 1000 kilometres and can take anywhere between a few days to several weeks to complete (Johnson and Groot, 1963; Groot and Margolis, 1991; Quinn, 2005; Clark *et al.*, 2016). Many authors suggest that during this time, smolts do not feed extensively and instead primarily use endogenous energy stores (Stefansson *et al.*, 2003; Quinn, 2005; Hinch *et al.*, 2006), although it should be noted that direct empirical evidence is scant and the evidence that has been recorded is mixed (Larsson *et al.*, 2011). Indeed, endogenous energy stores of long-distance migrating smolts can approach very low levels (Rondorf *et al.*, 1985; Stefansson *et al.*, 2003). For example, Rondorf et al. (1985) found that whole body lipids of hatchery-origin Chinook salmon (*Oncorhynchus tshawytscha*) migrating in the Columbia River decreased from 4.3% to 1.4% (a 65% decline) during the ~700 km migration between the release site and the estuary and that survival was higher for fish that had higher percent body lipid at the time of their release (Rondorf *et al.*, 1985). Thus, endogenous energy levels may decrease to very low levels during freshwater migration.

The physiology and energetic condition of individual fish could control their performance during migration. Under typical migration conditions, migrating fish rely primarily on fats and proteins for fuel (Driedzic and Hochachka, 1978; Brett and Groves, 1979). Fish migrants also commonly use protein during periods of food deprivation (Persson *et al.*, 2018). However, during periods of starvation, protein catabolism can compromise muscle tissue and likely comes at the expense of performance (Moon and Johnston, 1980; Sullivan and Somero, 1983; Black and Love, 1986; Kiessling *et al.*, 1990; Martinez, 2003). Thus, prolonged swim performance should decline as protein content declines. Lipid levels of 1.4%–2.0% are thought to be the lowest levels possible, as the remaining lipids are likely structural phospholipids of cell walls and are essential for survival (Castledine and Buckley, 1980). Indeed, Pacific salmon smolts are rarely observed to have lipid levels lower than 2.0% in the wild (DFO unpublished data), suggesting that this may be a threshold for survival. Therefore, migration success seems likely to be positively related to body condition, especially lipid levels. Furthermore, both condition and ionoregulation could shift through time during migration (Berggren and Filardo, 1993; Bassett *et al.*, 2018) and affect swim performance and survival during initial transfer to saltwater. Thus, the duration of freshwater migration could also influence swim performance and survival upon transition to saltwater.

It has been proven challenging to link observed changes in physical or energetic condition during migration to performance or survival. Few studies have examined swim performance of salmon smolts in relation to condition (Bams, 1967; Snyder, 1980; Persson *et al.*, 2018). More have studied condition-dependent survival, but mostly with hatchery fish in a laboratory setting (LeBrasseur, 1969; Connolly and Petersen, 2003; Simpkins *et al.*, 2003, 2004; Ferguson *et al.*, 2010) with several attempting to infer condition and survival from natural settings (size: Ricker, 1954; Ward *et al.*, 1989; Henderson and Cass, 1991; proximate body condition: Gardiner and Geddes, 1980; Biro *et al.*, 2004; Finstad *et al.*, 2004). Thus, there is a need for robust laboratory studies of the relationship between condition, performance and survival of out-migrating wild salmon.

Here we quantified the relationship between freshwater migration duration, body condition, swim performance and survival in a wild population of migratory sockeye salmon (*O. nerka*) held in a laboratory environment. Specifically, we asked the following questions: (i) How does duration of freshwater migration impact swim performance and survival? (ii) What are the relationships between energetic and physical condition and (a) prolonged swim performance or (b) survival? We found that freshwater migration duration did not influence prolonged swim performance or survival. We compared energetic condition metrics (i.e. proximate body composition and derived energy values) and physical condition (i.e. size, weight and condition factor) and found that condition factor best predicted prolonged swim performance, whereas energy density and protein content were predictive of survival. The swim performance threshold occurred approximately five weeks before the survival threshold, suggesting that swim performance may be a more biologically relevant endpoint for predicting survival in wild juvenile salmon. As a result, condition factor could be a useful tool for understanding how changes to freshwater habitat, which impact fish condition, could affect survival during seaward migration and the early marine life stage of salmon.

## Methods

### Overall study approach

**Figure 1:**
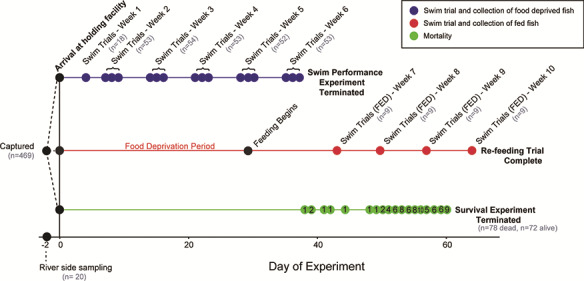
Experiment timeline, where fish were captured and a subset were sampled at the river (Day -2), before transportation to a holding site on Day 0. Fish were distributed evenly among three treatment groups (7D, 14D, 21D) and among three tanks within each group, for a total of nine tanks. Fish either were food deprived and completed a swim performance trial each week for 6 weeks (blue), held for 28 days before feeding for 2 weeks and then completed swim performance trial weekly for 4 weeks (pink) or held without food until death (green). Fish were randomly selected for swim trials across tanks from each treatment group for the swim performance experiment. A randomly selected subset from the 7D group were moved to a different tank and fed for the re-feeding trial. Grey ‘n’ indicates the number of different fish that completed the swim trial each week. Black ‘n’ indicates the daily mortality in the survival experiment.

We held juvenile sockeye salmon smolts without food for 61 days in three treatment groups modelled after freshwater migration durations of the Chilko Lake sockeye salmon population used in this study: (i) transferred to saltwater after 7 days, 7D group, which matches closely with average migration duration of Chilko Lake salmon smolts; (ii) transferred to saltwater after 14 days, 14D group, representing a delayed migration; and (iii) transferred to saltwater after 21 days, 21D group, representing a severely delayed migration (Clark *et al.*, 2016; Stevenson *et al.*, 2019) (Fig. 1). Different smolts from each of the three treatment groups completed a prolonged swim performance challenge each week for six weeks, until, for two successive weeks, >80% of fish could not finish the trial (swim performance endpoint). After the swim trials were completed, we continued to observe fish for mortality, and once half of the remaining fish being held had died (*n* = 78, survival endpoint) the experiment was terminated. After 28 days of food deprivation, we began feeding a subset of the 7D treatment group to monitor swim performance during recovery from starvation (*n* = 36). Approximately nine different fish from this fed group completed swim performance trials each week for five weeks to determine whether swim performance could be recovered after a period of food deprivation. The re-feeding experiment would ensure that energetic condition was the main factor that changed during holding and that a decrease in condition was related to a decrease in swim performance, rather than an artefact of holding time. We compared swim performance and survival between the three migration duration treatment groups and found no difference between groups. Since there was no difference between groups, we measured and calculated a variety of energy condition metrics (i.e. energy density, lipid content, moisture content, protein content and triglycerides (TAG)) for the 7D group, a subset of fish swam in experiment 1, and all the fish that died to determine the relationship between condition and prolonged swim performance (experiment 2) and survival (experiment 3).

### Fish collection

Wild juvenile sockeye salmon were caught during their seaward migration at the outlet of Chilko Lake (51.63 °N, 124.14 °W). Chilko Lake is an indicator population for management of Fraser River sockeye salmon and is one of the most productive sockeye salmon lakes in the Fraser River basin, producing an average of ~30 million sockeye salmon smolts annually (running average, since 1994). The predominate age class of Chilko Lake smolts is age 1; however, Chilko Lake produces some 2-year-old smolts typically ranging from 2% to 10% of the total outmigration cohort. Only 1-year-old fish (i.e. fish with fork lengths (FLs) of less than 110 mm) were used in this study. Migration takes 4–12 days as smolts travel the ~700 km from Chilko Lake through the Chilko, Chilcotin and Fraser Rivers to the Strait of Georgia (Clark *et al.*, 2016; Stevenson *et al.*, 2019). It takes an additional 30–45 days before salmon leave inland coastal waters after traversing the Strait of Georgia and Johnstone Strait (Clark *et al.*, 2016; Stevenson *et al.*, 2019). Previous work has shown that Chilko sockeye salmon smolts migrate at a rate of 30–220 km/day in freshwater and 10–25 km/day in the marine environment along the east coast of Vancouver Island (Welch *et al.*, 2009; Clark *et al.*, 2016; Stevenson *et al.*, 2019).

Juvenile sockeye salmon were captured by dip net on the evening of 3 May 2017 at the Chilko smolt enumeration fence, a full fence weir managed by Fisheries and Oceans Canada Fraser River Sockeye Stock Assessment Program. Twenty fish were euthanized on site with an overdose of MS222 (0.5 g/L) and preserved at −80 °C for energetic analysis. Remaining fish were held overnight in an aerated 1000-L holding tank (5.8–7.5 °C; 80.1%–109.9% O_2_ saturation) and the next day were driven ~12 hr to the holding facility at Simon Fraser University in Burnaby, BC. The present study was conducted in accordance with the Canadian Council on Animal Care, as administered by Simon Fraser University (1238B-17).

### Fish holding

Smolts were divided into the three treatment groups, 7D (*n* = 179), 14D (*n* = 180) and 21D (*n* = 182), and were randomly assigned to three tanks per treatment, thus sockeye salmon smolts were held in nine oval holding tanks (200 L) for up to 64 days (61 days for survival experiment, 64 days for fed treatment; 4 May–7 July 2017; Fig. 1) at a density of <2 smolts per litre. Water was flushed through the tanks at a rate of >2 L per minute. Holding tanks were covered with plexiglass, half of which was black to allow for shading in the tanks. A single air stone was used to enhance oxygen saturation, which never decreased below 90% in any tank (Handy Polaris, OxyGuard Internation A/S, Farum, Denmark). Smolts were originally held in dechlorinated and UV-sterilized freshwater and were transitioned to saltwater at the three respective time points (7D, 14D, 21D). Fish in each group were transitioned gradually over 36 hrs to saltwater at a similar concentration to what they would experience during migration through the Fraser River estuary and coastal Vancouver Island (28–30 ppt; Thomson, 1981). Saltwater was made to a concentration of 28–30 ppt (Instant Ocean® Sea Salt) and allowed to sit for 24 hrs before being added to the circulation system. As it was being recirculated among tanks, saltwater was filtered through carbon filters. Approximately 30% of saltwater was replaced every 2 to 3 days to keep ammonia concentrations below 2 ppm. Water temperature was held constant with a mean of 10.7 °C (7.8–12.9 °C) consistent with ambient May/June Fraser River water temperature (MacDonald *et al.*, 2019). A water pump temporarily went out on 6 June 2017 and water temperature increased in all tanks to 13–14.5 °C for ~14 hrs, no mortalities were recorded resulting from that event and 14.5 °C is within the range of temperatures experienced by smolts in the natural environment (MacDonald *et al.*, 2019). Smolts were held in 12:12 hr light:dark conditions.

Holding tanks were checked at least twice daily. Dead fish were removed, FL (mm) and weight (g) were recorded and fish were frozen at −80 °C for later analysis. Any distressed fish, characterized by gasping at the water surface or loss of equilibrium, were euthanized with an overdose of MS222 (0.5 g/L). Food was withheld from fish in the nine tanks until approximately half of the fish had died. Remaining fish were euthanized with an overdose of MS222 (0.5 g/L). All fish were measured, weighed and frozen at -80 °C for later analysis.

### Re-feeding

After 28 days of food deprivation, we transferred a subset of 36 smolts from the 7D group to a 150-L holding tank and began feeding them (7D-fed) twice daily with commercial pellets (EWOS Canada Ltd Surrey, Canada) in excess (~2% body weight). Nine different fed fish were swum each week after 2, 3, 4 and 5 weeks of feeding.

### Swim flume

Prolonged swim performance tests were completed in a fixed velocity flow-through swim flume (14-cm wide, 25-cm tall, 240-cm long; a plexiglass insert was used to narrow the swim arena to 9-cm wide, 15-cm tall, 142-cm long to increase water velocity), where water was pumped into the front of the flume, flowed through a honeycomb structure to increase laminar flow, and through a mesh net out the back into a recirculating tank. At the farthest end of the flume, a vertically sliding plexiglass door could be adjusted to ensure the water level in the flume was at 15 cm. A black plastic cover provided shade in the centre of the flume and lights in front and behind the shaded region were used to encourage fish to swim in the shaded middle of the swimming arena where the flow was most consistent. Flow was measured at the top, middle and bottom of the water column in both salt and freshwater.

**Table 1:** Changes in sockeye salmon smolt morphometrics, energy density and proximate body constituents during the experiment for fish collected at Chilko River sampling site, for fish from the 7-day saltwater transfer group and for fish that had died during experiment

	River sampling	Week 1		Week 2^a^		Week 3		Week 4		Week 5		Week 6		Deaths^b^
Completed trial? (Y/N)	NA	N	Y	N	Y	N	Y	N	Y	N	Y	N	Y	NA
Sample size	20	3	15	11	7	9	6	13	2	16	1	13	1	78
FL (mm) (SE)	87.2 ±0.9	83.0 ±1.5	86.7 ±1.3	82.5 ±1.6	87.3 ±1.5	84.4 ±1.5	84.0 ±2.4	86.9 ±1.6	86.0 ±1.0	85.2 ±1.2	88.0	86.4 ±1.3	85.0	81.8 ±0.6
Weight (g) (SE)	4.91 ±0.15	4.84 ±0.09	4.97 ±0.22	3.72 ±0.23	4.55 ±0.26	3.86 ±0.25	3.82 ±0.34	3.97 ±0.21	4.31 ±0.26	3.63 ±0.17	4.14	3.68 ±0.16	3.87	2.81 ±0.06
Fulton’s condition factor (SE)	0.74 ±0.01	0.85 ±0.06	0.76 ±0.02	0.65 ±0.01	0.68 ±0.01	0.63 ±0.01	0.64 ±0.01	0.60 ±0.01	0.68 ±0.17	0.58 ±0.01	0.61	0.57 ±0.01	0.63	0.51 ±0.01
Energy density (MJ/kg) (SE)	4.29 ±0.06	4.39 ±0.06	4.27 ±0.08	4.85 ±0.12	4.60 ±0.07	4.44 ±0.09	4.49 ±0.21	3.96 ±0.06	4.37 ±0.20	4.00 ±0.08	4.18	3.83 ±0.06	4.00	2.82 ±0.02
Water (% wet weight) (SE)	78.77 ±0.20	77.89 ±0.24	77.92 ±0.27	74.62 ±0.57	75.48 ±0.33	76.39 ±0.42	76.34 ±0.70	78.51 ±0.25	77.20 ±0.29	77.82 ±0.41	76.74	78.49 ±0.25	77.87	82.96 ±0.15
Protein (% wet weight) (SE)	16.14 ±0.15	17.12 ±0.17	17.49 ±0.14	19.68 ±0.49	19.22 ±0.29	18.13 ±0.31	17.91 ±0.56	16.40 ±0.23	17.59 ±0.04	16.76 ±0.36	18.49	15.86 ±0.22	17.05	11.32 ±0.10
Lipid (% wet weight) (SE)	2.88 ±0.16	2.62 ±0.25	2.09 ±0.15	2.46 ±0.21	2.04 ±0.11	2.21 ±0.15	2.45 ±0.32	1.84 ±0.10	2.31 ±0.58	1.77 ±0.05	1.29	1.78 ±0.09	1.57	1.49 ±0.03
TAG (% lipid) (SE)	24.9 ±2.6	46.7 ±9.8	39.6 ±4.3	28.8 ±4.3	21.8 ±3.6	17.4 ±1.7	14.2 ±4.3	16.9 ±1.3	41.3^c^ ±6.5	15.1^c^ ±1.3	NA	16.9^c^ ±1.4	24.6	21.1^c^ ±1.1

^a^Fish were transferred to saltwater 3 days before Week 2 sampling/swim performance trials.

^b^Deaths that occurred before survival endpoint was reached.

^c^One TAG sample was removed because a precipitate formed, thus TAG was calculated from a n-1 sample size for that group.

### Prolonged swimming experiment

Of the three types of swim performance tests (burst swim performance (<2 min), prolonged swim performance (<120 min) or critical swim speed tests (>120 min); Beamish, 1978), we chose to perform prolonged swimming trials as they are rooted in biological realism and are related to fish condition and health (Beamish, 1978). Prolonged swim performance affects migratory capacity as well as the ability of fish to capture prey and evade predators, and thus is linked to survival and fitness (Plaut, 2001).

Smolts from each of the three groups underwent a swim performance trial each week for six weeks to determine if swim performance differed between treatments groups (experiment 1) as well as across differing physical or energetic condition (experiment 2). A total of 6 swim trials were performed every week (2 trials for each of the 3 treatment groups), with 9 fish in each trial (18 from each of the 3 treatment groups), for a total of ~54 fish swum each week. The swim trials in the first week were an exception in which, due to time constraints, we swam six fish from each group randomized over two trials, and across all holding tanks. These fish were unmarked and were not assigned to a treatment group, therefore they provide a baseline swim performance for all groups, but were excluded from swim trial analysis. Fish were always swum in water of the same condition (i.e. salinity, temperature, oxygen saturation) as they were being held. Fish from each treatment group were randomly assigned to each swim trial, when possible given different holding salinities. For example, during the second week, only the 7D group fish were in saltwater so they were swum in two trials (nine 7D fish in each trial), whereas both the 14D and 21D groups were in freshwater and were therefore randomly assigned to four trials (four to five fish from the 14D and 21D groups in each trial). Two trials were completed each day, with the order of trials randomized each week. Each trial consisted of two swim performance tests, one in the morning and one repeat test in the afternoon on the same fish to assess repeatability of performance. Repeat swim performance data are available in the supplemental information (Fig. S1). The evening before each swim trial, 18 fish were removed from their holding tanks and individually marked using a fin clip. Fish were anaesthetized in MS222 (0.05 g/L), and the tips of the ventral and/or anal fins were clipped, enabling unique identification of each fish. Fish were recovered overnight in two 150-L aquarium with 9 fish in each tank. For fish that were being fed, food was withheld 24 hrs before the swim trial was completed. We chose to cease swim trials after 6 weeks as >80% of the different fish swum in each trial could not finish the first test in 2 consecutive weeks (weeks 5, 6; swim performance endpoint).

For each test, 9 individually marked fish were placed in the swim flume for a 12 min acclimation period at a flow rate of ~0.085 m/s (~1 BL/s). Following the acclimation period, flow rate was steadily increased to 0.50 m/s (4.5–6.4 BL/s) over a period of 12 min. Fish that did not swim before the flume reached full flow were gently prodded three successive times with a blunt instrument to encourage swimming. If they still refused to swim, they were removed from the study. Once at 0.50 m/s, flow rate was held constant for 90 min (pilot swim tests in week 1 confirmed 90 min was required to achieve variability in swim performance). Fish that fell to the mesh net at the back of the flume were gently prodded three successive times with a blunt instrument to encourage swimming. If they remained at the back mesh, the time was recorded to the nearest 10 sec and the fish was removed from the flume and placed in a glass aquarium for the remainder of the trial. Fish were allowed to rest for at least two hours before the swim test was repeated. After completing the second swim test, fish were euthanized with an overdose of MS222 (0.5 g/L). Fish were weighed (g), FL (mm) was measured and fish were frozen on dry ice and stored at −80 °C until analyses.

### Body condition analyses

Of the fish that participated in the swim performance trials, we only measured the energetic condition of fish from the 7D (*n* = 179) and 7D-fed (*n* = 36) groups, because all 3 unfed treatments (7D, 14D and 21D) had similar swim performance and the 7D group was most biologically consistent with natural migration duration/timing of Chilko Lake sockeye smolts. Physical condition was assessed for all treatment groups by measuring FL (mm) and wet mass (g) and by calculating Fulton’s condition factor (equation 1). We determined proximate body composition (moisture, lipid and protein content) for all fish that died during, or were alive at the end of the experiment, as well as the fish from the 7D and 7D-fed groups that underwent the swim performance trials (Table 1). Carbohydrate content was not determined as food was withheld prior to collection, and carbohydrates such as muscle and liver glycogen are not a significant source of stored energy in fish (Brett, 1995), rather lipid and protein are the main fuel used by fish during periods of food deprivation (Driedzic and Hochachka, 1978; Brett and Groves, 1979; McKeown, 1984). We decided to measure TAG, in addition to all lipid content, as TAG is the main form of stored lipids in vertebrates (Driedzic and Hochachka, 1978), and therefore their depletion could represent a decrease in energy condition (Driedzic and Hochachka, 1978; Cleary *et al.*, 2012).

Proximate body analysis was completed according to methods of Crossin (2003) that were adapted from Higgs *et al.* (1979) and Bligh and Dyer (1959). Briefly, whole fish were homogenized in a SPEX SamplePrep 2010 Geno/Grinder (SPEX, Metuchen, NJ) at 1500 rpm for 2-min. intervals until completely homogenized. A sample of 0.3 g plus/minus symbol as in percent water and ash sections 0.015 g of homogenate was weighed for each of lipid, water and ash analysis. Together, lipid, water and ash percentages can be used to calculate protein content and energy density.

Lipid extraction: A mixture of methanol, chloroform and water in ratio of 1:1:0.48 was added to the sample and homogenized. Samples were filtered with a Büchner funnel and the supernatant was decanted into a graduated cylinder. Once biphasic layers of chloroform–lipid and methanol–water formed, the volume of the lipid–chloroform layer was measured and the top water–methanol solution was aspirated away. A 100-μL sub-sample of the lipid–chloroform layer was removed and stored at −80 °C for determination of TAG concentration. The remaining chloroform–lipid solution was pipetted into pre-weighed aluminium boats, and the remaining lipid was weighed when chloroform had completely evaporated. All samples were analysed in duplicate, and only samples that differed by less than 0.5% were retained. Lipid values are reported in percent lipid of total fish wet mass.

Percent water: Whole body moisture content was determined by drying a 0.3 g ± 0.015 g sample of homogenate in an oven overnight (16–20 hr) at 100 °C. Samples were then placed in a desiccator for 15 min and weighed. All samples were analysed in duplicate, and only replicates that differed by less than 1.5% were retained. Body moisture values are reported in percent water of total fish wet mass.

Ash: A 0.3 g ± 0.015 g sample of each homogenate was transferred to the furnace and combusted at 600 °C for 2.5 hrs. Samples were placed in a desiccator for 15 min and then weighed a final time. All samples were analysed in duplicate, and only samples that differed by less than 0.5% were retained.

**Table 2:** Model comparison for generalized linear models predicting swim success/failure for 7D group

	K	Log likelihood	∆AIC	ωAIC
Condition factor	2	−44.07	0.00	0.71
Condition factor + TAG	3	−44.03	2.06	0.25
TAG + weight	3	−46.14	6.27	0.03
Weight	2	−48.83	9.52	0.01
TAG + FL	3	−51.38	16.76	0.00
TAG	2	−52.48	16.82	0.00
Protein content + FL	3	−53.83	21.67	0.00
Energy density + FL	3	−53.89	21.77	0.00
Protein content	2	−55.25	22.37	0.00
Energy density	2	−55.32	22.50	0.00
Intercept	1	−57.29	24.34	0.00
Lipid content	2	−56.65	25.16	0.00
FL	2	−56.68	25.21	0.00
Lipid content + FL	3	−55.78	25.55	0.00
Moisture content	2	−56.98	25.82	0.00
Moisture content + FL	3	−55.97	25.93	0.00

TAG = percent triglycerides of lipid, FL = fork length (mm), ∆AIC = difference in AIC between models, ωAIC = Akaike weight of model. FL, weight and condition factor were determined from fish before they were frozen; *n* = 90.

**Table 3:** Model comparison for generalized linear models predicting swim failure/death for all treatment groups combined

	K	Log likelihood	∆AIC	ωAIC
Energy density	2	0.00^a^	0.00	0.37
Protein content	2	0.00^a^	0.00	0.37
Protein content + FL	3	0.00^a^	2.09	0.13
Energy density + FL	3	0.00^a^	2.09	0.13
Moisture content	2	−4.70	9.39	0.00
Moisture content + FL	3	−4.64	11.38	0.00
Lipid content	2	−59.93	119.87	0.00
Lipid content + FL	3	−59.86	121.82	0.00
Condition factor + TAG	3	−68.84	139.80	0.00
Condition factor	2	−76.52	153.04	0.00
TAG + FL	3	−81.48	165.07	0.00
TAG	2	−83.33	166.67	0.00
TAG + weight	3	−82.85	167.82	0.00
Weight	2	−90.46	180.91	0.00
Intercept	1	−91.73	181.40	0.00
FL	2	−90.84	181.67	0.00

^a^Energy density and protein content of fish separate perfectly allowing for probabilities of 0 or 1. As a result model estimates of confidence intervals approach infinity.

TAG = percent triglycerides of lipid, FL = fork length , ∆AIC = difference in AIC between models, ωAIC = Akaike weight of model. FL, weight and condition factor were determined from fish before they were frozen; *n* = 133.

Triglyceride: TAG concentration was determined using a colorimetric assay kit (Cayman Chemicals, Ann Arbour, MI, # 10010303). Briefly, the 100-μL sub-samples of the lipid–chloroform layer were thawed and chloroform was evaporated using nitrogen gas (15 PSI). Samples were reconstituted with isopropanol, vortexed for 15 sec and incubated at room temperature for 1 hr. Ten microlitres of either TAG standard (0, 3.125, 6.25, 12.5, 25, 50, 100, 200 mg/dl) or sample were assayed in duplicate. Note that the standard was prepared with isopropanol. A chloroform blank, in which 100 μL of chloroform was evaporated and reconstituted with isopropanol, was also used to ensure chloroform did not affect absorbance. Average absorbance was measured at 530–550 nm using a FLUOstart Omega multimode microplate reader (BMG Labtech, Ortenberg, Germany). Final TAG values were reported in percent TAG of lipid (g TAG/g lipid*100). TAG samples from 5 fish were excluded from analyses (2 from 7D group: 1 from week 4 and 1 from week 5; 2 from 7D-fed group in week 9; and 1 that died during the experiment), as they developed a precipitate when added to the isopropanol and the solids interfered with absorbance, leading to an overestimate of TAG concentration.

Calculations: We calculated smolt condition parameters:

Fulton’s condition factor (K) was calculated using equation (1):

(1) \begin{equation*} K=\frac{Wt}{FL^3}\ast 100, \end{equation*}

where weight ($Wt$) is in grams and fork length ($FL$) is in centimetres.

The percent of whole body protein ($P$) was calculated from percentages of water ($W$), lipids ($L)$ and ash$(A)$ (Hendry *et al.*, 2000) using equation (2):

(2) \begin{equation*} P=100-\left(W+L+A\right). \end{equation*}

Energy density (D; MJ/kg) can be calculated from the amount (g/kg) of lipid (*l*) and protein (*p*) using equation (3):

(3) \begin{equation*} D=l\ast 0.0362+p\ast 0.0201, \end{equation*}

where 0.0363 and 0.0201 are the energy densities of lipid and protein, respectively (Brett and Groves, 1979).

### Statistical analyses

Survival analysis was used to compare prolonged swim performance or survival among the three treatment groups: 7D, 14D, 21D. Failure time, or survival analysis, is used to compare groups of right-censored data (Therneau and Grambsch, 2000). Swim trials were a form of right-censored data, as trials were ended after 90 min and thus a fish could have continued to swim for 1 min or several more hours and this information was lost. A Cox proportional hazards model was used to determine significance of the effect of treatment group on swim time or survival. Analysis of variance was used to compare each physical variable (i.e. FL, weight and condition factor) across 7D, 14D and 21D treatment groups to determine if variables differed across groups. Post hoc Bonferroni pairwise tests were used to compare physical and energetic condition variables between fish that were dead or alive at the end of the survival experiment (Table S3). We calculated a Bonferroni correction (0.05/8 tests) and applied a significance level of α = 0.00625.

We used generalized linear models to test specific hypotheses of the relationship between energetics and swim performance and survival. Use of a generalized linear model resulted in some loss of data precision [data were compressed to either ‘completed’ trial (finished 90 min swim trial) or ‘failed’ trial (swam for less than 90 min of swim trial), resulting in loss of individual swim time], at the benefit of using a model that has an established method for use in prediction. We used a generalized linear modelling approach, fit with a binomial distribution and a logit link function, to compare models of different correlated smolt condition variables and determine the most parsimonious models of swimming success/survival. No random effect was used as the number of fish that completed the swim trial was very small in some weeks, which prevented convergence. However, top model residuals were examined for patterns among rearing tanks and weeks, and none were found, suggesting tank and week did not affect model output (Figs S2 and S3). For the swim performance model, we used energetic correlates from the 7D group only, since swim performance for the 7D, 14D and 21D groups did not differ. Therefore, we compared eight individual-level variables for the 7D group (i.e. weight, length, condition factor, energy density, percent lipid, percent water, percent protein and TAG) in 16 models (Table 2). For the survival model, we used energetic variables from all three groups combined and compared 16 models, as with the swim performance model (Table 3). All variable combinations, except those that were highly correlated (correlation coefficient, >0.6; Fig. S4) or not independent (i.e. condition factor was not independent for length or weight, since it is calculated from length and weight), were used in models. All variables were standardized and centred. Model comparison was completed using Akaike Information Criterion corrected for small sample sizes (AIC_c_), where models with <2$\Delta$AIC were considered most parsimonious (Burnham and Anderson, 2002). The top swim performance model was validated using k-fold validation, whereby the model was re-parameterized with 90% of the data (training dataset) and used to predict the remaining 10% of data (test dataset). Predicted probabilities of ≥0.5 were considered to have completed the swim test and <0.5 were considered to have failed the swim test. Predictions were compared to observations to determine model predictive performance. This procedure was repeated 1000 times with samples randomly assigned to either training or test datasets. All statistics were performed in R statistical computing environment (v 3.5.2) (R Core Team, 2018) using RStudio GUI (v1.1.463, 2018) and the following packages AICcmodavg (Mazerolle, 2017), lme4 (Bates *et al.*, 2015) survival (Therneau, 2015) and coxme (Therneau, 2020), and graphing was done with ggplot2 (Wickham, 2009).

**Figure 2:**
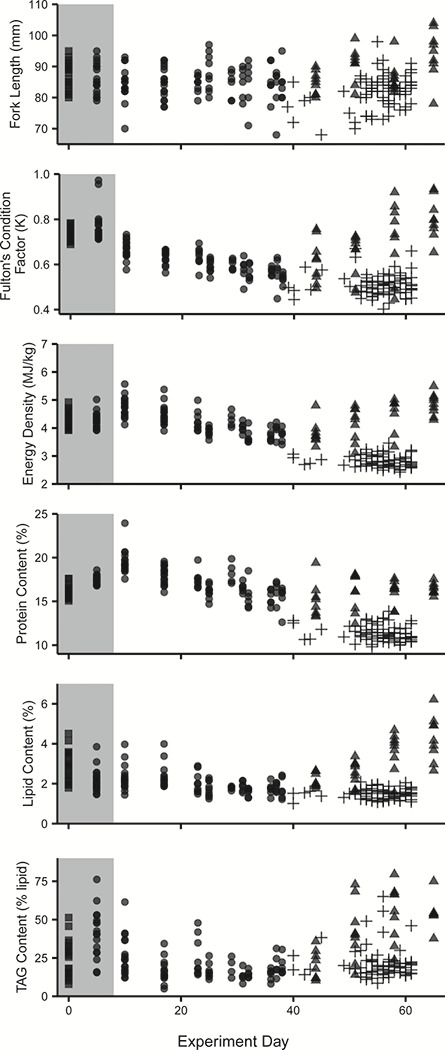
Physical and energetic condition variables changed throughout the holding period for individuals that were measured immediately after being caught (square), held without food and alive (7D group only; closed circle), held without food and died (all treatment groups; cross) and fed after 28 days of starvation (7D-fed group; triangle). Shaded region represents fish sampled in freshwater, non-shaded region represents fish held in saltwater. Generally, condition metrics decreased through time. Protein content and energy density (calculated from protein and lipid content) increased after transfer to saltwater, likely due to short-term dehydration during the physiologic transition to life in seawater.

**Figure 3:**
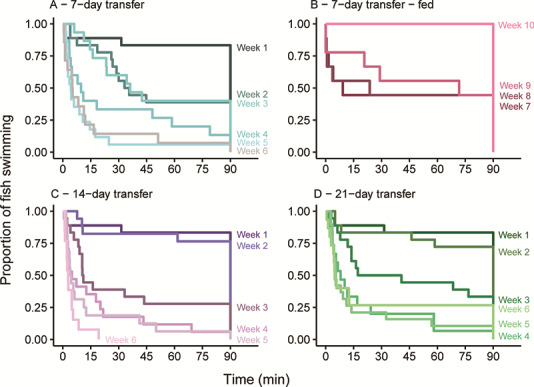
Swim performance (time swimming up to 90 min) of fish held without food and transferred to saltwater after (**A**) 7 days, (**C**) 14 days and (**D**) 21 days and (**B**) fish that were transferred to saltwater after 7 days, food deprived for 28 days before beginning feeding in week 5 such that week 7 represents two weeks of feeding, week 8 represents three weeks of feeding and so forth. Each shade of a colour represents a week of swim trials, which were conducted weekly.

**Figure 4:**
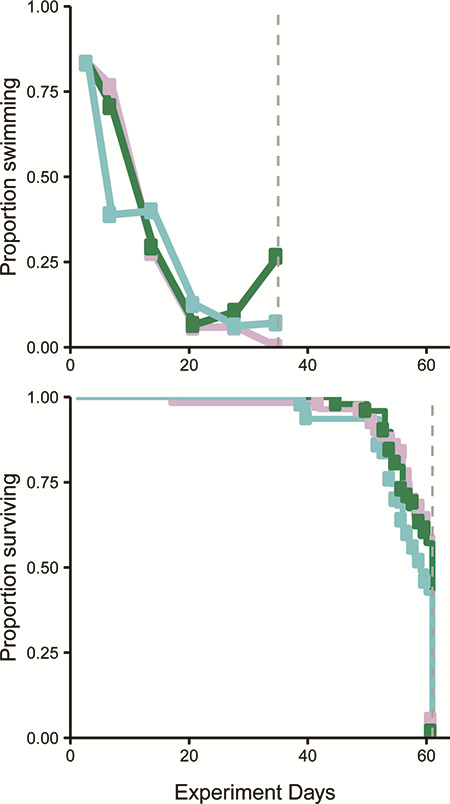
Weekly proportion of food-deprived fish that completed the 90 min swim trial (top) or survived (bottom) for each treatment group, transferred to saltwater after 7 days (blue), 14 days (light purple) and 21 days (green). Grey vertical line represents the end of the testing/observation period. Observation periods ended after >80% of the fish could not finish the test for over two weeks (top) and after 50% of fish had died (bottom).

**Figure 5:**
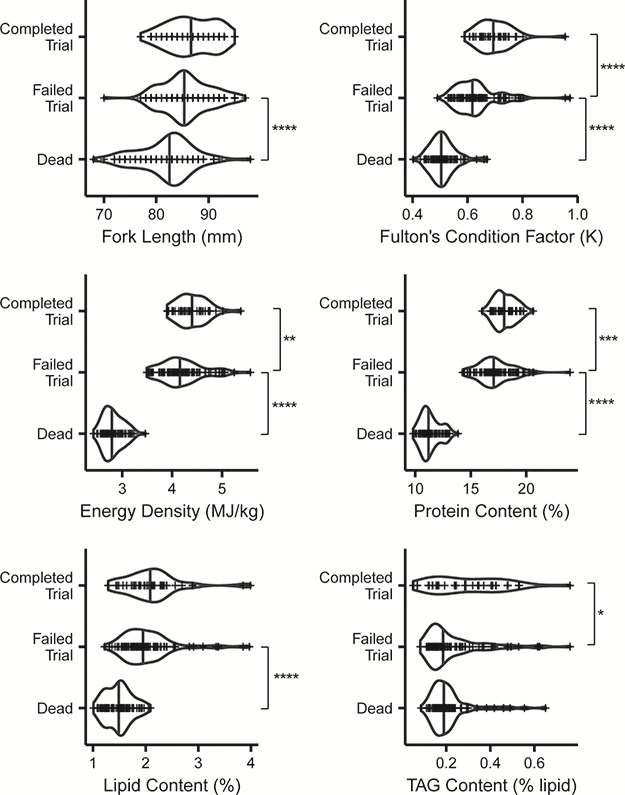
Swim performance and survival by energetic correlates for starved fish. Completed, fish that finished the 90 min swim; Failed, fish that did not complete the 90 min swim; and Dead, fish that died during the survival experiment. This analysis groups all weeks of swimming together. Significance of post hoc Bonferroni pairwise tests are indicated by either ^*^P ≤ 0.05, ^**^P ≤ 0.01, ^***^P ≤ 0.001 or ^****^P ≤ 0.0001.

## Results

### Food deprivation and individual attributes

Wild sockeye salmon smolts kept for up to 61 days without food decreased in condition factor, energy density and lipid and protein content, and increased in water content throughout the period of food deprivation. Between collection in the river and termination of the experiment (61 days), smolts (both alive and dead at the end of the experiment) lost an average of 1.74 g (35% of initial body weight), resulting in a 28% decrease in Fulton’s condition factor (K) (0.74 to 0.53) and a 34% decrease in energy density (4.29 MJ/kg to 2.83 MJ/kg). Lipid values decreased on average from 2.88% to 1.49%, and protein content decreased from 16.14% to 11.41%. TAG did not change, shifting from 25% of lipid to 22% of lipid.

Shifts in body energy metrics and condition factor were due to the experimental treatments of food deprivation rather than an artefact of holding, given that fish condition metrics returned to pre-experiment levels within 3 to 4 weeks for the 7D-fed group that were food deprived for 28 days (Fig. 2). Over the 28 days of re-feeding, 7D-fed fish almost doubled their initial average weight of 3.63 g in week 5, growing to an average of 6.89 g and had begun to grow in length (~10 mm), which led to a 60% increase in Fulton’s condition factor (0.5 to 0.8). Fish also recovered energetic condition with a 20% increase in energy density (4.00 MJ/kg to 4.84 MJ/kg) due to an increase in lipid content (1.77% to 4.28%), as protein content stayed relatively stable. TAG levels also increased from 15% in week 5 at the start of feeding to 54% TAG of lipid (Table S2).

### Experiment 1: migration duration and swim performance/survival

Swim performance decreased equally for all unfed groups with >80% of fish not being able to finish the swim test after greater than four weeks of food deprivation (based on 283 fish in 32 trials). Twenty-one smolts (7.4%) would not swim in the flume, instead falling to the back of the flume and remaining there during the acclimation period (Table S1). These fish were removed from the study. Fish that were included in the swim trials swam actively in the flow, and generally did not exhibit any ‘cheating’ behaviours such as swimming near the front of the flume where current was more irregular or drifting behind another fish. More fish completed the trial in week 1 (83%), compared to week 6 (12%) (Figs 3 and 4). When swim performance was compared across the three treatment groups (timing of switch to saltwater) using Cox proportional hazards (weeks 2–6, week 1 was not included in this analysis), the hazard rate (i.e. effect size) was not significantly affected by group (Wald test = 1.35, df = 2, *P* = 0.5). Swim trials were terminated in week 6, because the swimming endpoint had been exceeded (more than two successive weeks where >80% of fish failed the trial). Fish were held for an additional three weeks, until half of the remaining fish had died (50% mortality, survival endpoint). There were no differences in mortality across all three groups (Wald test = 1.92, df = 2, *P* = 0.4). Despite a decrease in overall physical and energetic condition no mortalities occurred during or close to the transfer of smolts to saltwater. Groups did not differ in FL (ANOVA, df = 2, F-value = 0.895, *P* = 0.41) and weight (ANOVA, df = 2, F-value = 2.956, *P* = 0.05).

### Experiment 2: prolonged swimming and condition

Concomitant with a decrease in swim performance, condition decreased throughout the swim trials. Since swim performance did not differ among groups, we determined the relationship between swim performance and condition metrics using data from the 7D group (7D, *n* = 108; Table 1). Over the 6 weeks that the swim trials were completed, smolts from the 7D transfer group lost an average of 1.25 g (25% of initial body weight), resulting in a 26% decrease in Fulton’s condition factor (K) (0.77 to 0.57) and 11% decrease in energy density (4.29 MJ/kg to 3.83 MJ/kg). Lipid values decreased on average from 2.20% to 1.74%, protein from 17.42% to 15.94% and TAG from 41% of lipid to 17% of lipid (Table 1, Table S4).

**Figure 6:**
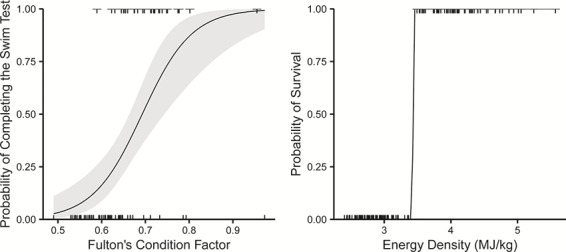
Top model predicted probabilities of 7D fish completing the swim trial (1 = complete, 0 = incomplete; left) and top model predicted probabilities of fish failing the swim test (1) or dying (0) for all un-fed groups (right). Grey shaded regions represent 95% confidence regions.

Grouped across weeks, fish that completed the swim test had 14% higher condition factor (0.71 complete vs. 0.61 incomplete), higher energy density (4.39 MJ/kg complete vs. 4.20 MJ/kg incomplete) and higher protein, lipid and TAG content, compared to those that did not complete the swim test (Fig. 5). We compared generalized linear models using AIC_c_ to determine which set of energetic variables best predicted whether fish completed the 90 min swim test or failed the test (stopped swimming before 90 min) (Tables 2 and 4). Energetic variables were highly correlated (Fig. S4). We found that the most parsimonious model predicting completion of the swim test included only condition factor as a predictor variable (∆AIC < 2; Fig. 6, left). This model was followed by one that included condition factor and TAG (∆AIC = 2.06). We used k-fold model validation to determine that the top model accurately predicted whether a fish would pass or fail the swim test ~79.5% of the time.

Swim performance rebounded in 7D-fed group confirming that the manipulation of energy levels was the determinant of changes in swim performance. When fed for 21 days, subsequent to 28 days of food deprivation, swim performance increased (57% percent of fish finished the swim trial after 3 weeks of feeding); however, swim performance did not immediately return to pre-starvation levels. It took ~3 weeks of feeding for energetic correlates to return to or increase above capture levels, suggesting some effects of either holding or legacy effects from starvation (Table S2). After 35 days (week 10) of feeding 100% of fish completed the test, however fish had begun growing which could have affected the results of the test (Fig. 3, Table S2).

### Experiment 3: survival and condition

Mortality during the 61-day holding period was biased towards the end of the experimental period, with the majority of fish dying in the last 10 days of the experiment. At the termination of the survival experiment, 78 fish had died while 72 fish remained alive and were euthanized. Fish that remained alive at the end of the experiment were slightly larger than fish that died during the experiment (FL = 86.3 mm vs. 81.8 mm: Bonferroni *t*-test, *P* > 0.0001; weight = 3.58 g vs. 2.81 g: Bonferroni *t*-test, *P* > 0.0001, respectively; Table S3, Fig. S5). Energy density and proximate body constituents were not markedly different. Fish at the end of the experiment were likely close to death, which makes it difficult to compare energy metrics between those alive or dead at the termination of the experiment. Therefore, it is more useful to compare fish condition of fish that failed to swim (i.e. fish that could not complete the swim trial, but were still alive at the swim performance endpoint) and fish that died by the end of the experiment (Table 1). Energy density and protein content values between fish that failed the swim trial and fish that died during the experiment separated nearly completely (did not overlap), thus linear models with these covariates were predicted perfectly (Fig. 4) and were the best fit models (Tables 3 and 4). For example, the range of energy density values for dead fish was 2.40–3.46 MJ/kg, whereas the range of values for a fish that did not swim was 3.49–5.57 MJ/kg. Similarly, the range of protein for fish that died before the end of the experiment was 9.36–14.13% and 14.27–23.94% for fish that failed to swim. Thus, thresholds for fish surviving based on energy density and/or protein were 3.47 MJ/kg and 14.20%.

## Discussion

We held sockeye salmon smolts for 61 days during which we observed swim performance and survival and found that a continuous decline in condition factor was predictive of swim performance, while a threshold existed between energy density/protein content and survival. We chose two experimental endpoints (swim performance: 80% of fish could not finish the first test; survival: 50% of the fish had died) to represent both a biologically relevant and more clinical endpoint, respectively. The first endpoint, a lack of swimming capacity, represents a more ecologically relevant endpoint than a clinical diagnosis of death because fish with decreased swim performance likely have a higher probability of being predated (Plaut, 2001). The second endpoint can be used to compare with other starvation studies and represents a ‘true’ clinical endpoint, where outside of external factors such as predation, a fish would die. There was no difference in swim performance or survival between treatment groups (7D, 14D, 21D) suggesting that freshwater migration duration did not influence these two metrics. Using swim performance data from the 7D group as a response variable and physical or energetic condition predictor variables in a generalized linear model framework, we found that Fulton’s condition factor was the best predictor variable of the swim performance endpoint. Indeed, k-fold validation indicated that using Fulton’s condition factor alone could predict whether a fish could successfully complete a 90-min prolonged swim trial 79.5% of the time. We also compared the group of fish that could not finish the swim trial to the group of fish that died and found an energy density threshold of 3.47 MJ/kg below which a fish will die. The swim performance endpoint was reached at higher energy levels, and well before the survival endpoint was reached. The swim performance endpoint was observed in individuals with energy levels close to the lowest levels observed in natural systems (Rondorf *et al.*, 1985; D. Patterson, personal communication), indicating that swim performance and therefore condition factor, may be a more accurate representation of survival estimates in natural populations.

Contrary to our prediction, condition factor, rather than energetic variables, was the best predictor of swim performance. Swim performance decreased as condition factor decreased. Length and condition factor are low effort, low cost and non-lethally collected variables, which are often measured in field studies and have been related to smolt survival (Healey, 1982; Ward *et al.*, 1989; Henderson and Cass, 1991; Duffy and Beauchamp, 2011). This is not the first study to find that condition factor rather than energetic variables or metabolites better predicted swim performance in fishes (Atlantic cod *Gadus morhua*, Martinez, 2003; largemouth bass *Micropterus salmoides*, Gingerich *et al.*, 2010; gilthead seabream *Sparus aurata*, Faria *et al.*, 2011). Here we demonstrate this relationship in salmon smolts.

Energetic variables were the best predictors of survival. Interestingly, TAG was not the best predictor of either swim performance or survival, and in fact appeared stable throughout the experiment. Energy density (energy from lipid and protein) and lipid content have been related to survival in salmon and other species (LeBrasseur, 1969; Simpkins *et al.*, 2003, 2004; Ferguson *et al.*, 2010; Persson *et al.*, 2018), and since TAG is a non-structural lipid, we predicted that this variable would better predict survival compared to lipid content. However, energy density and protein content were most distinct between the fish that failed the swim test and the fish that died, rather than lipid or TAG. The importance of energy density, rather than lipid content, is likely due to the inter-individual variance in use of protein and lipid as energy storage (McCue, 2010). It is also possible that variables other than what we measured could have better predicted swim performance and/or survival. For example, metabolic rate, metabolic enzymes, organ mass (e.g. liver somatic index) and glycogen have been correlated with condition and performance (Martinez, 2003, 2004; Gingerich *et al.*, 2010). However, in these previous studies the fish were not starved and the utility of these measurements likely changes during starvation (McCue, 2010). Alternatively, fatty acids could be correlated with performance as Pierce *et al.* (2005) found that dietary fatty acid influenced performance in a migratory bird. Thus, future research could clarify the roles of different components of energy stores in performance and survival.

Energy values observed in our experiment were similar to what has been observed in natural systems and other studies. The median lipid content for those that failed the trial was 1.93%, approaching values previously estimated to be the lower threshold for survival in the wild (DFO, unpublished data). Indeed, wild sockeye salmon smolts have rarely been found in the wild with lipid values lower than 2.0% or condition factors less than 0.57 (DFO, unpublished data). This study examined fish just prior to migration, and so likely represents fish with reserved energy for migration. Rondorf *et al*. (1985) found that lipid values approached 1.4% and energy density decreased to 1.02 Kcal/g (4.27 MJ/kg) for hatchery origin Columbia River Chinook salmon smolts nearing the estuary after completion of freshwater migration. The energy density values found in their study fall close to the group of fish that did not complete the swim trial in our experiment, whereas the lipid values fall closer to those that died during our experiment. Persson *et al.* (2018) found a threshold for survival of 3.5 MJ/kg and condition factor of 0.65 in starved Atlantic salmon (*Salmo salar*) smolts, consistent with our findings. Another study held juvenile chum salmon (*O. keta*) captured in the Icy Strait, AK, USA, without food for 45 days in saline water in living-stream tanks and found that energy density (determined using a bomb calorimeter) decreased to ~650 cal/g (2.72 MJ/kg) (Ferguson *et al.*, 2010), similar to the lowest energy densities measured in fish held for 61 days in our experiment (Table S3). At the end of our experiment, surviving fish were longer than dead fish (Fig. S1), which is consistent with Simpkins et al. (2004) who found that for sedentary fish held without food, smaller fish died before larger fish, perhaps due to decreased lipid stores. Together with the swimming results, this suggests that smaller, lower condition fish might be more likely to die under conditions of starvation (Biro *et al.*, 2004). Furthermore, these examples could represent species- or population-specific differences in physiological response to starvation. Indeed, swim performance differs across populations of fed coho (*O. kisutch*) and sockeye salmon (Taylor and McPhail, 1985; Eliason *et al.*, 2017). However, our findings that fish with energy densities of <3.47 MJ/kg are unlikely to survive appear consistent with other literature (Rondorf *et al.*, 1985; Persson *et al.*, 2018).

**Table 4:** Top model parameter coefficients for generalized linear models predicting swim success/failure and swim failure/death

Top model	Parameter	Coefficient (95% CI)
(Success/failure) = condition factor		
	Intercept	−1.62 (−2.38, −0.97)
	Condition factor (K)	1.68 (0.95, 2.56)
(Swim failure/death) = energy density		
	Intercept	−1299^a^
	Energy density	379^a^
(Swim failure/death) = protein content		
	Intercept	−4601^a^
	Protein content	324.1^a^

^a^Energy density and protein content of fish separated perfectly allowing for probabilities of 0 or 1. As a result model estimates of confidence intervals approach infinity.

Food-deprived fish held in the laboratory setting likely lost energy more slowly than non-feeding fish that would be actively migrating. Fish have very low resting metabolic rates (10–30 times less than mammals and up to 100 times less than birds of the same weight), such that the majority of energy is spent on locomotion (Brett, 1972). Fish held in the laboratory moved very little, and thus likely spent much less energy per unit time than an actively foraging or migrating fish. In fact, Rondorf et al. (1985) found that salmon smolts held in the laboratory lost ~15% body lipids versus 65% in fish that had been released and were actively migrating over the same period. In a more recent study of hatchery-origin rainbow trout (*O. mykiss*), sedentary fish (held in tanks with no directional current) had percent lipid values that averaged 3.5% higher than active fish (in tanks with directed current, forcing fish to swim) held without food for the same period of time (Simpkins *et al.*, 2003). Therefore, caution needs to be taken when considering the real-world application of our laboratory-based longevity estimates. Hence, our finding of an 80% failure rate after four weeks of starvation is likely a substantial overestimate compared to what would be observed in natural settings, with actual time to 80% failure being much less than four weeks, depending on water temperature. Similarly, we would expect that fish in a natural setting would likely die much earlier than the 51–61 days in which we observed the majority of mortality in our experiment. Longevity in the natural environment is likely less than the 3–6 weeks in which we observed fish complete swim trials, yet juvenile salmon migrations in altered rivers can take over two weeks to complete (Giorgi *et al.*, 1997; Smith *et al.*, 2002).

Both aerobic prolonged swim performance and anaerobic burst swim performance are likely related to the ability to capture prey, escape predators and migrate (Plaut, 2001). We measured prolonged swim performance, as an indicator of endurance, and found a marked decrease during starvation that is consistent with existing literature (Martinez, 2003) and with our original hypothesis. Decreased metabolic and contractile capabilities of fish muscle tissue during starvation was the likely underlying mechanism for the observed decrease in swim performance (Moon and Johnston, 1980; Sullivan and Somero, 1983; Black and Love, 1986; Kiessling *et al.*, 1990; Martinez, 2003). An additional component of swim performance that was not measured was anaerobic (burst) swim performance, which is used to quickly escape from predators or capture prey. Existing literature suggests that anaerobic swim performance may be more impacted than aerobic performance during starvation (Beardall and Johnston, 1983; Lowery and Somero, 1990; Martinez, 2004), and thus represents a future avenue for research. Therefore, aerobic swim performance as measured in this study represents a conservative indicator of the response to a decrease in condition.

Our study is one of a few studies that examines swim performance and survival in a wild fish. Others have used hatchery fish, due to their availability, readiness to live in aquaculture facilities and ease of access. However, swim performance of hatchery fish can be much lower than that of wild fish (Bams, 1967; Beamish, 1978; McDonald *et al.*, 1998; Simpkins *et al.*, 2003, 2004; Pedersen *et al.*, 2008). For example, we used the same apparatus as Collins et al. (2013), which examined prolonged swim performance of hatchery-reared sockeye salmon. The majority of fish in their experiment tired by 20 min; however, we required a 90-min swim to achieve variability in swim performance in the first week of the swim trials. McDonald et al*.* (1998) made a similar observation when they compared swim performance of hatchery reared and wild Atlantic salmon. Wild fish had better fin quality, higher anaerobic capacity and higher swim endurance than hatchery fish (McDonald *et al.*, 1998). Hatchery fish may differ from wild fish which makes it challenging to draw conclusions about swim performance of wild fish and velocity barriers in natural systems, using hatchery-reared fish (McDonald *et al.*, 1998).

Understanding condition-dependent swim performance and survival could help elucidate carry-over effects and limits on survival during seaward and early marine migration in Pacific salmon. Survival through the early marine life history stage is size-dependent, and likely condition-dependent, as juvenile salmon must grow quickly to escape gape-limited predators and expand prey options (Pope *et al.*, 1994). Schooling behaviours may decrease predation risk through ‘predator swamping’, but these schooling behaviours require some level of swim performance (Furey *et al.*, 2021). In fact, predation may be condition-dependent, where lower-condition individuals are preferentially predated (Tucker *et al.*, 2016). Thus, condition-dependent swim performance relationships could help predict probability of survival in natural systems. Additionally, most of the freshwater migration and some fraction of the marine migration corridor is food-limited (McKinnell *et al.*, 2014), and therefore condition-dependent swim performance and survival relationships could clarify individual and population-level sensitivity to starvation.

Using wild salmon smolts and an experimental approach, we have developed a model to predict swim performance given condition factor. Condition factor is a simple and inexpensive tool that represents a likely avenue for measuring swim performance and thus survival probability. Indeed, prolonged swim performance collapsed three weeks prior to and at higher energy levels than the onset of mortalities. Survival was better predicted by energy density and protein content than condition factor. Energy density is a more clinical predictor of survival and is a more labour intensive and expensive measurement and requires lethal sampling. Given that different individuals and populations of salmon have different energetic status and condition (MacDonald *et al.*, 2019), or may use estuary stopover habitat for different durations (Moore *et al.*, 2016), here we provide the foundation for understanding the impacts of this variation. This study offers a new tool for contextualizing population-specific sensitivity to changes in migratory conditions or pre-migratory conditioning phase.

## Funding

This work was supported by Vanier Canadian Graduate Scholarship, Weston Family Scholarship awarded to S.M.W. Additional funding was from the Liber Ero Foundation for J.W.M. [421208] and support from Pacific Salmon Commission Southern Endowment Fund and DFO Environmental Watch Program.

## Supplementary Material

Limits_on_salmon_migration_-_supplement_R1_coab014
