# Supplementary material for: Limits on performance and survival of juvenile sockeye salmon (Oncorhynchus nerka) during food deprivation: a laboratory-based study
Source: Conserv Physiol. 2021 Mar 24;9(1):coab014. doi: 10.1093/conphys/coab014 (PMC8009553; doi:10.1093/conphys/coab014)
Supplement: Limits_on_salmon_migration_-_supplement_R1_coab014 [file limits_on_salmon_migration_-_supplement_r1_coab014.docx]

# Supplemental

## Supplemental Tables

Table S1: Morphometrics and condition factor for sockeye salmon smolts from each treatment group swum each week in swim trials.

|  |  | Week 1 | Week 2 | Week 3 | Week 4 | Week 5 | Week 6 |
| --- | --- | --- | --- | --- | --- | --- | --- |
| All groups |  |  |  |  |  |  |  |
|  | Sample size^1^ | 18 | NA | NA | NA | NA | NA |
|  | Num. did not swim | 0 | NA | NA | NA | NA | NA |
|  | Fork Length (mm) (SE) | 86.1 ± 0.8 | NA | NA | NA | NA | NA |
|  | Weight (g) (SE) | 4.95 ± 0.13 | NA | NA | NA | NA | NA |
|  | Fulton’s Condition Factor (SE) | 0.77 ± 0.01 | NA | NA | NA | NA | NA |
|  | Median swim time (min) | 90.0 | NA | NA | NA | NA | NA |
|  | Percent completed swim (%) | 83 | NA | NA | NA | NA | NA |
| 7-day SW transfer |  |  |  |  |  |  |  |
|  | Sample size^1^ | NA | 18 | 15 | 15 | 17 | 14 |
|  | Num. did not swim | NA | 0 | 3 | 2 | 1 | 4 |
|  | Fork Length (mm) (SE) | NA | 84.4 ± 1.0 | 84.3 ± 0.9 | 86.8 ± 1.0 | 85.4 ± 0.9 | 86.3 ± 1.0 |
|  | Weight (g) (SE) | NA | 4.04 ± 0.16 | 3.85 ± 0.15 | 4.01 ± 0.13 | 3.66 ± 0.13 | 3.69 ± 0.13 |
|  | Fulton’s Condition Factor (SE) | NA | 0.66 ± 0.01 | 0.63 ± 0.01 | 0.61 ± 0.01 | 0.58 ± 0.01 | 0.57 ± 0.01 |
|  | Median swim time (min) | NA | 34.4 | 36.2 | 9.8 | 4.4 | 5.2 |
|  | Percent completed swim (%) | NA | 39 | 40 | 13 | 6 | 7 |
| 14-day SW transfer |  |  |  |  |  |  |  |
|  | Sample size^1^ | NA | 17 | 18 | 17 | 15 | 13 |
|  | Num. did not swim | NA | 0 | 0 | 1 | 0 | 4 |
|  | Fork Length (mm) (SE) | NA | 86.1 ± 1.0 | 86.1 ± 1.2 | 84.3 ± 1.0 | 86.6 ± 1.1 | 83.6 ± 1.5 |
|  | Weight (g) (SE) | NA | 4.67 ± 0.13 | 4.26 ± 0.19 | 3.71 ± 0.16 | 3.85 ± 0.13 | 3.29 ± 0.17 |
|  | Fulton’s Condition Factor (SE) | NA | 0.73 ± 0.01 | 0.65 ± 0.01 | 0.61 ± 0.01 | 0.59 ± 0.01 | 0.56 ± 0.01 |
|  | Median swim time (min) | NA | 90.0 | 10.8 | 4.9 | 3.9 | 2.9 |
|  | Percent completed swim (%) | NA | 76 | 28 | 6 | 7 | 0 |
| 21-day SW transfer |  |  |  |  |  |  |  |
|  | Sample size | NA | 18 | 18 | 15 | 19 | 15 |
|  | Num. did not swim | NA | 0 | 0 | 3 | 0 | 3 |
|  | Fork Length (mm) (SE) | NA | 85.8 ± 0.81 | 86.8 ± 0.78 | 85.8 ± 1.17 | 86.0 ± 0.73 | 87.3 ± 1.01 |
|  | Weight (g) (SE) | NA | 4.65 ± 0.12 | 4.52 ± 0.10 | 3.84 ± 0.16 | 3.80 ± 0.11 | 3.91 ± 0.16 |
|  | Fulton’s Condition Factor (SE) | NA | 0.73 ± 0.01 | 0.69 ± 0.01 | 0.60 ± 0.01 | 0.59 ± 0.01 | 0.58 ± 0.01 |
|  | Median swim time (min) | NA | 90.0 | 29.0 | 8.0 | 5.4 | 4.8 |
|  | Percent completed swim (%) | NA | 72 | 33 | 7 | 11 | 27 |

^1^Sample size is the number of fish that swam in the study. Mean fork length, weight and Fulton’s condition factor, and median swim time were calculated from fish that swam only. The fish that did not swim during initial acclimation phase of swim performance trial were removed from the study.

Shaded values indicate values taken from fish that were in freshwater at time of swim trial.

Table S2: Changes in sockeye salmon smolt morphometrics, proximate body constituents, and energy density during experiment for fish from the 7-day saltwater transfer group that were fed after 28 days of food deprivation.

|  | Week 7^1^ | | Week 8 | | Week 9 | | Week 10 | |
| --- | --- | --- | --- | --- | --- | --- | --- | --- |
| Completed Trial? (Y/N) | N | Y | N | Y | N | Y | N | Y |
| Sample size^2^ | 4 | 4 | 3 | 4 | 3 | 4 | 0 | 9 |
| Fork Length (mm)  (SE) | 83.5  ±2.0 | 87.0  ±1.5 | 88.0  ±3.5 | 93.8  ±1.9 | 84.0  ±1.2 | 88.8  ±3.3 | NA | 94.1  ±2.6 |
| Weight (g)  (SE) | 3.25  ±0.24 | 4.60  ±0.47 | 4.39  ±0.72 | 5.89  ±0.42 | 4.48  ±0.30 | 5.66  ±0.61 | NA | 6.89  ±0.71 |
| Fulton’s Condition Factor  (SE) | 0.56  ±0.03 | 0.70  ±0.04 | 0.63  ±0.04 | 0.71  ±0.01 | 0.76  ±0.08 | 0.80  ±0.03 | NA | 0.80  ±0.03 |
| Energy Density (MJ/kg)  (SE) | 3.66  ±0.04 | 4.21  ±0.30 | 4.38  ±0.36 | 4.38  ±0.12 | 4.69  ±0.10 | 4.91  ±0.03 | NA | 4.84  ±0.13 |
| Water (% wet weight)  (SE) | 80.07  ±0.03 | 77.88  ±1.29 | 77.57  ±1.35 | 77.70  ±0.49 | 76.69  ±0.29 | 76.24  ±0.25 | NA | 76.95  ±0.25 |
| Protein (% wet weight)  (SE) | 14.13  ±0.43 | 17.04  ±1.29 | 16.50  ±1.35 | 17.02  ±0.49 | 16.42  ±0.29 | 16.95  ±0.25 | NA | 16.38  ±0.25 |
| Lipid (% wet weight)  (SE) | 2.27  ±0.22 | 2.16  ±0.16 | 2.93  ±0.24 | 2.65  ±0.20 | 3.85  ±0.27 | 4.16  ±0.23 | NA | 4.28  ±0.38 |
| TAG (% lipid)  (SE) | 20.8  ±3.7 | 24.6  ±7.0 | 49.7  ±9.3 | 48.2  ±8.8 | 54.0  ±7.6 | 66.4  ±5.8 | NA | 54.4^3^  ±4.0 |

^1^Fish were fed starting experiment week 5, therefore week 7 represents 14 days of feeding, week 8 represents 21 days of feeding, week 9 represents 28 days of feeding and week 10 represents 35 days of feeding.

^2^Only counts fish that swam during experiment, some fish refused to swim in tunnel (1 in week 7, 2 in weeks 8 and 9) and were removed from experiment.

^3^Two triglyceride (TAG) samples were removed as it was cloudy/precipitate had formed and lipid extraction could not be repeated.

Table S3: Condition and energetic variables at the end of the experiment for individuals that had died during the experiment or for individuals still alive at the end of the experiment

|  | Deaths | Alive at end of experiment |
| --- | --- | --- |
| Sample size | 78 | 72 |
| Fork Length (mm)  (SE) | 81.8  ±0.6 | 86.3*  ±0.5 |
| Weight (g)  (SE) | 2.81  ±0.06 | 3.58*  ±0.07 |
| Fulton’s Condition Factor  (SE) | 0.51  ±0.01 | 0.55*  ±0.01 |
| Energy Density (MJ/kg)  (SE) | 2.82  ±0.02 | 2.85  ±0.03 |
| Water (% wet weight)  (SE) | 82.96  ±0.16 | 83.54*  ±0.14 |
| Protein (% wet weight)  (SE) | 11.33  ±0.10 | 11.49  ±0.13 |
| Lipid (% wet weight)  (SE) | 1.49  ±0.03 | 1.49  ±0.02 |
| TAG (% lipid)  (SE) | 21.1  ±1.1 | 22.7  ±1.1 |

**^*^**indicates statistical difference (pairwise Bonferroni t-test) between dead and alive at the end of the experiment at α=0.00625, adjusted for increased false positive errors associated with multiple statistical tests.

Table S4: P values of pairwise tests between condition at capture and condition during subsequent periods of the experiment.

| Capture | Week 1 | Week 2 | Week 3 | Week 4 | Week 5 | Week 6 | End of Experiment (alive and dead) |
| --- | --- | --- | --- | --- | --- | --- | --- |
| Fork Length  (mm) | 1.0 | 1.0 | 1.0 | 1.0 | 1.0 | 1.0 | 0.01 |
| Weight  (g) | 1.0 | 1.0 | 0.08 | **0.0001** | **<0.0001** | **<0.0001** | **<0.0001** |
| Fulton’s Condition Factor | 1.0 | 1.0 | **<0.0001** | **<0.0001** | **<0.0001** | **<0.0001** | **<0.0001** |
| Energy Density (MJ/kg) | 1.0 | **0.002** | 1.0 | 1.0 | 0.4 | **0.0004** | **<0.0001** |
| Water  (% wet weight) | 1.0 | **<0.0001** | **<0.0001** | 1.0 | 1.0 | 1.0 | **<0.0001** |
| Protein  (% wet weight) | 0.02 | **<0.0001** | **<0.0001** | 1.0 | 1.0 | 1.0 | **<0.0001** |
| Lipid  (% wet weight) | 0.01 | 0.9 | 0.08 | **<0.0001** | **<0.0001** | **<0.0001** | **<0.0001** |
| TAG  (% lipid) | **0.0005** | 1.0 | 0.5 | 1.0 | 0.6 | 1.0 | 0.2 |

**Bold** indicates statistical difference (pairwise Bonferroni t-test) at α=0.00625, adjusted for increased false positive errors associated with multiple statistical tests.

Table S5: P values of pairwise tests between condition at the start of feeding (Week 5) and condition during subsequent periods of the experiment for 7D-fed fish.

|  | Week 7 | Week 8 | Week 9 | Week 10 |
| --- | --- | --- | --- | --- |
| Fork Length  (mm) | 1.0 | 0.1 | 1.0 | **0.0013** |
| Weight  (g) | 1.0 | 0.1 | 0.2 | **<0.0001** |
| Fulton’s Condition Factor | 1.0 | 0.7 | **0.002** | **<0.0001** |
| Energy Density (MJ/kg) | 1.0 | 0.7 | **0.002** | **<0.0001** |
| Water  (% wet weight) | 1.0 | 1.0 | 0.3 | 0.06 |
| Protein  (% wet weight) | 1.0 | 1.0 | 1.0 | 1.0 |
| Lipid  (% wet weight) | 1.0 | 0.1 | **<0.0001** | **<0.0001** |
| TAG  (% lipid) | 1.0 | **0.0007** | **<0.0001** | **<0.0001** |

**Bold** indicates statistical difference (pairwise Bonferroni t-test) at α=0.00625, adjusted for increased false positive errors associated with multiple statistical tests.

## Supplemental Figures

| 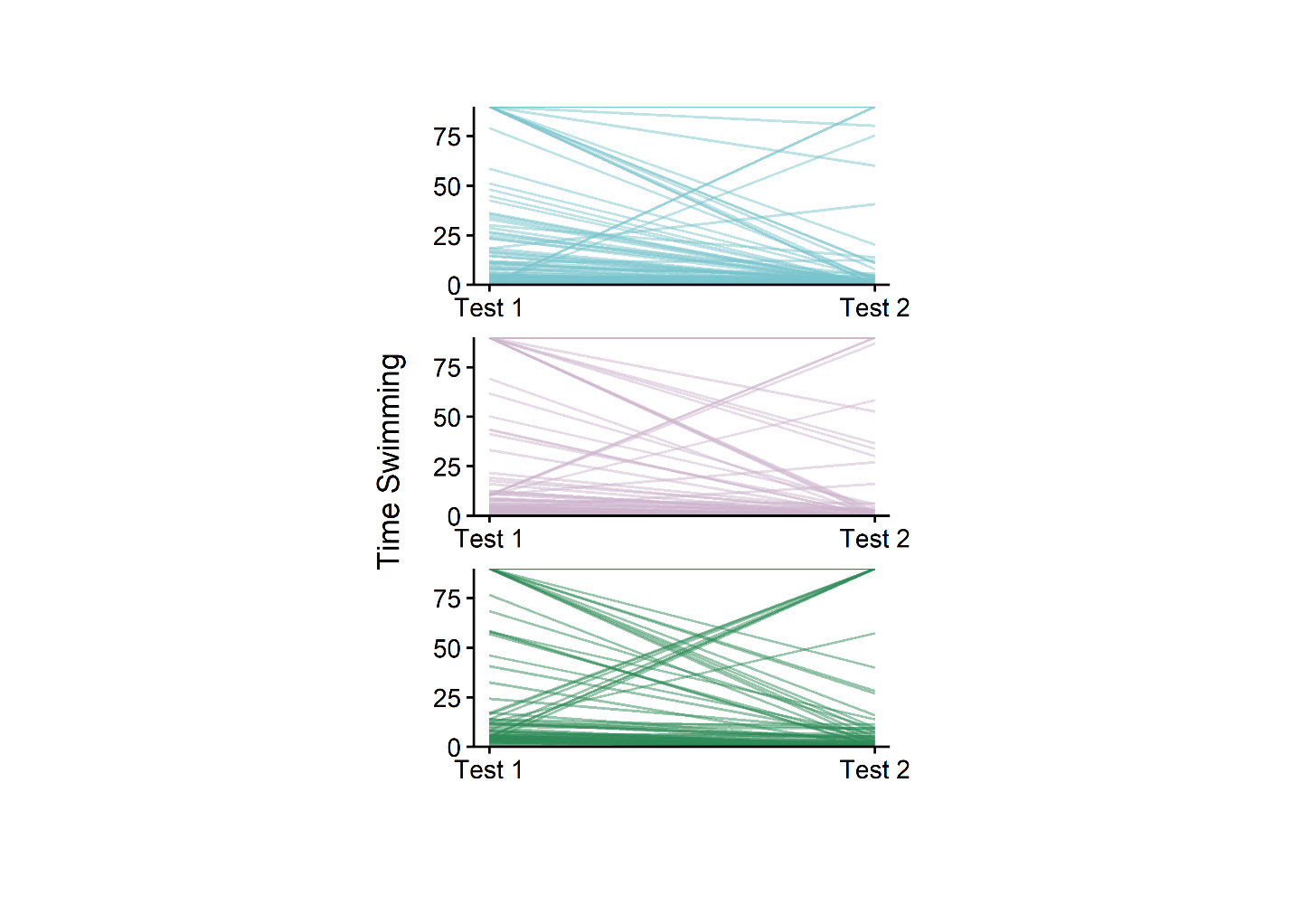 |
| --- |
| Fig S1: Repeatability of swim performance tests for all non-fed groups (7-day transfer (blue; top), 14-day transfer (light purple; middle), and 21-day transfer to saltwater (green; bottom)). Generally, fish swam for less time in test 2 than in test 1. |

| 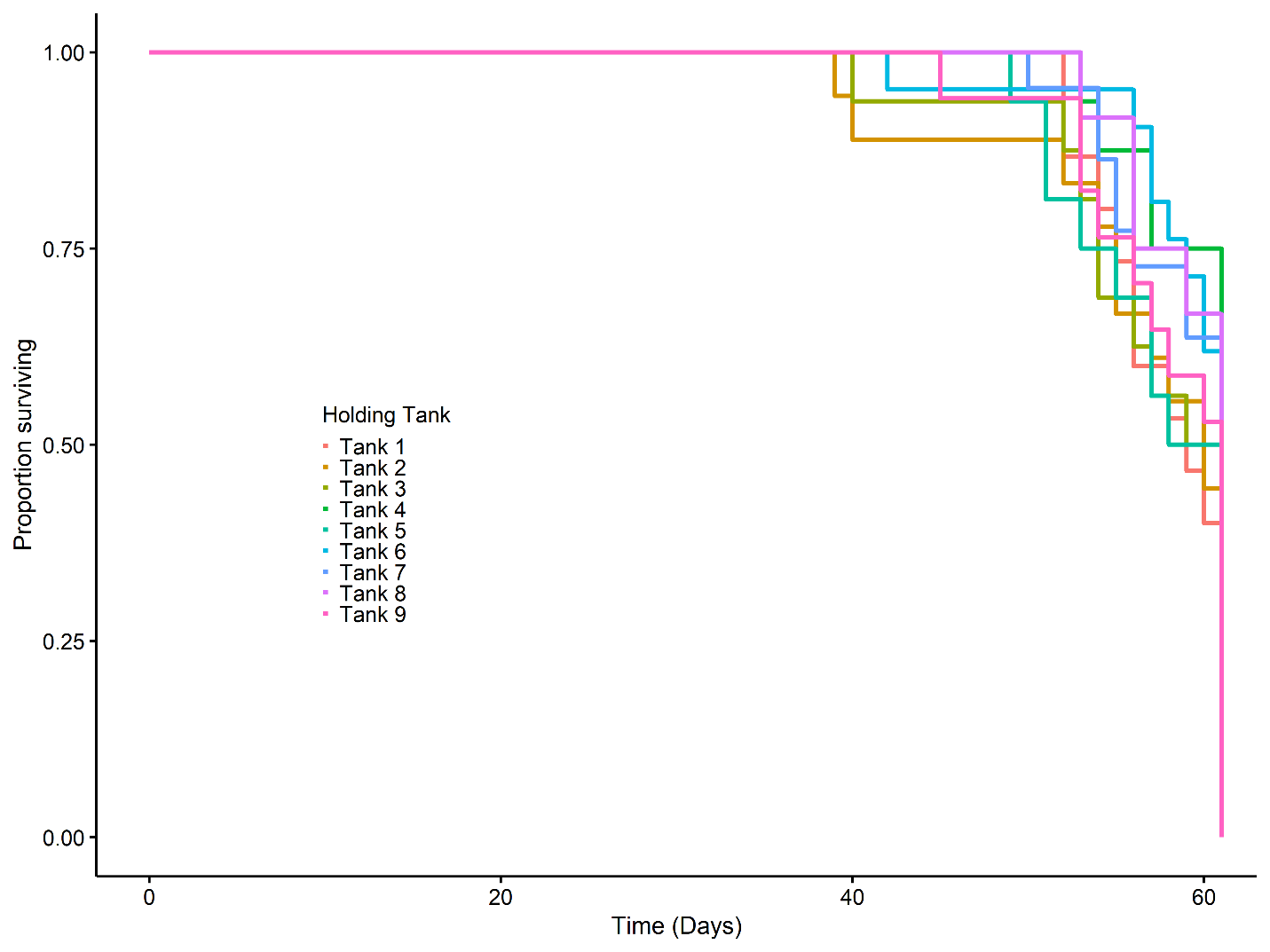 |
| --- |
| Fig S2: Survival curve for fish, broken down by holding tank. Fish were transferred to saltwater 7 days (holding tanks 1,2,3), 14 days (holding tanks 4,5,6) and 21 days (holding tanks 7,8,9) after emigration from their rearing lake. |

| 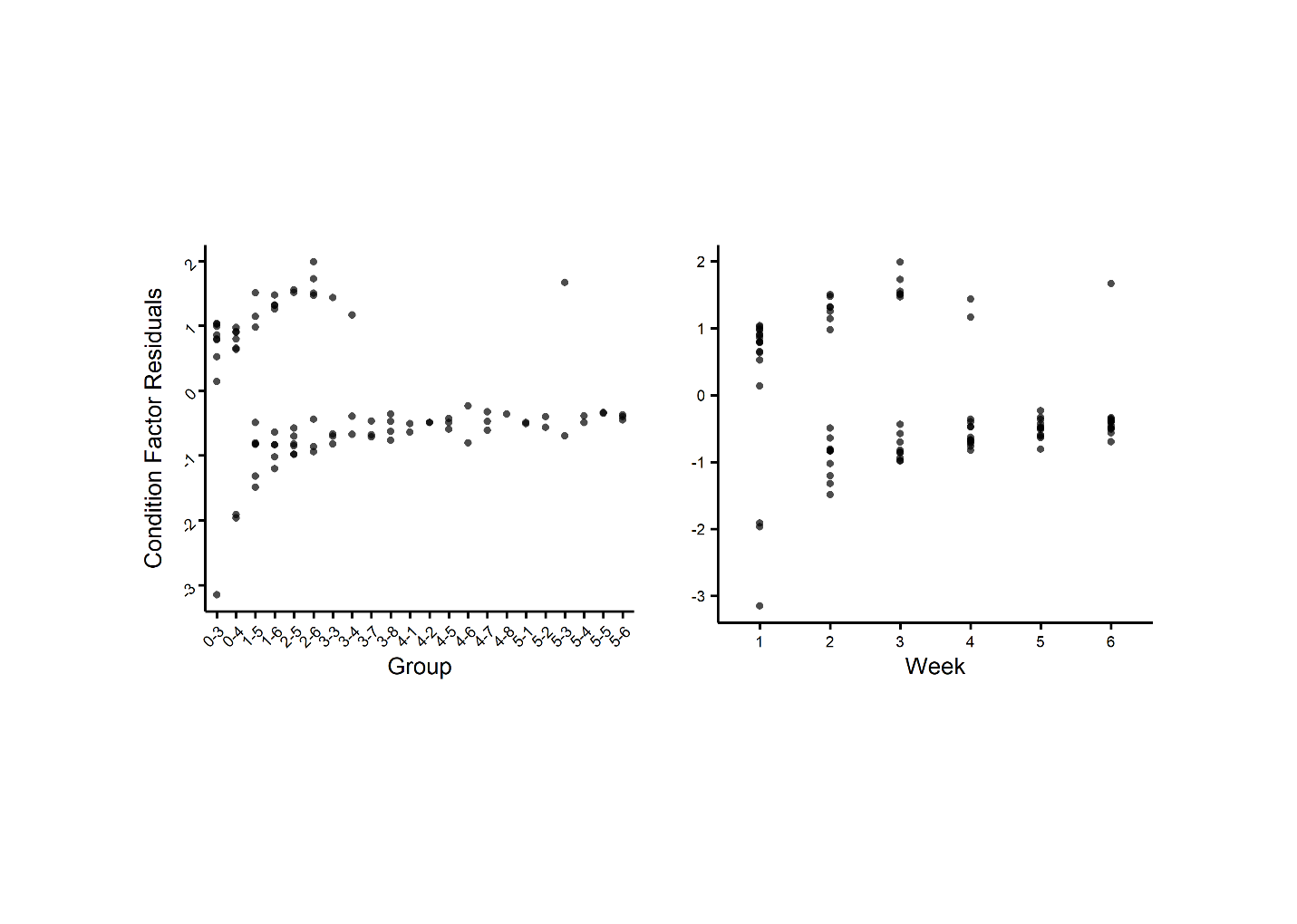 |
| --- |
| Fig S3: Residuals from the generalized linear model predicting completion or failure of the swim trial using condition factor compared to swim group or experimental week. Due to limited number of individuals that completed swim trials in later weeks, group and week could not be added as a random effect. Here we demonstrate that there is no clear pattern in residuals across groups and weeks of the experiment. Group designation is indicated by ‘week’-‘trial group’, where trial group was designated between 1 – 6. We completed ANOVAs comparing condition factor residuals and either group or week and found no significant effect of either (Group, df = 21, F value = 0.531, p = 0.947; Week, df = 5, F value = 1.325, p = 0.262). |

| 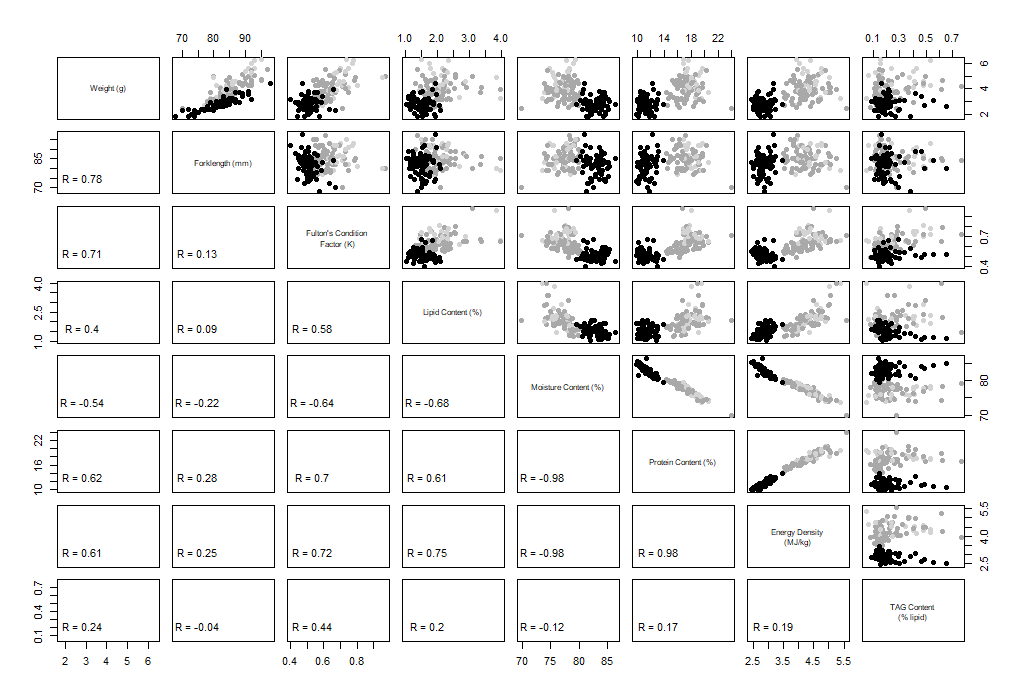 |
| --- |
| Fig S4: Correlation of condition metrics. Black points are for those that died during the experiment, dark grey for those that could not finish the swim trial, and light grey for those that finished the swim trial. |

| 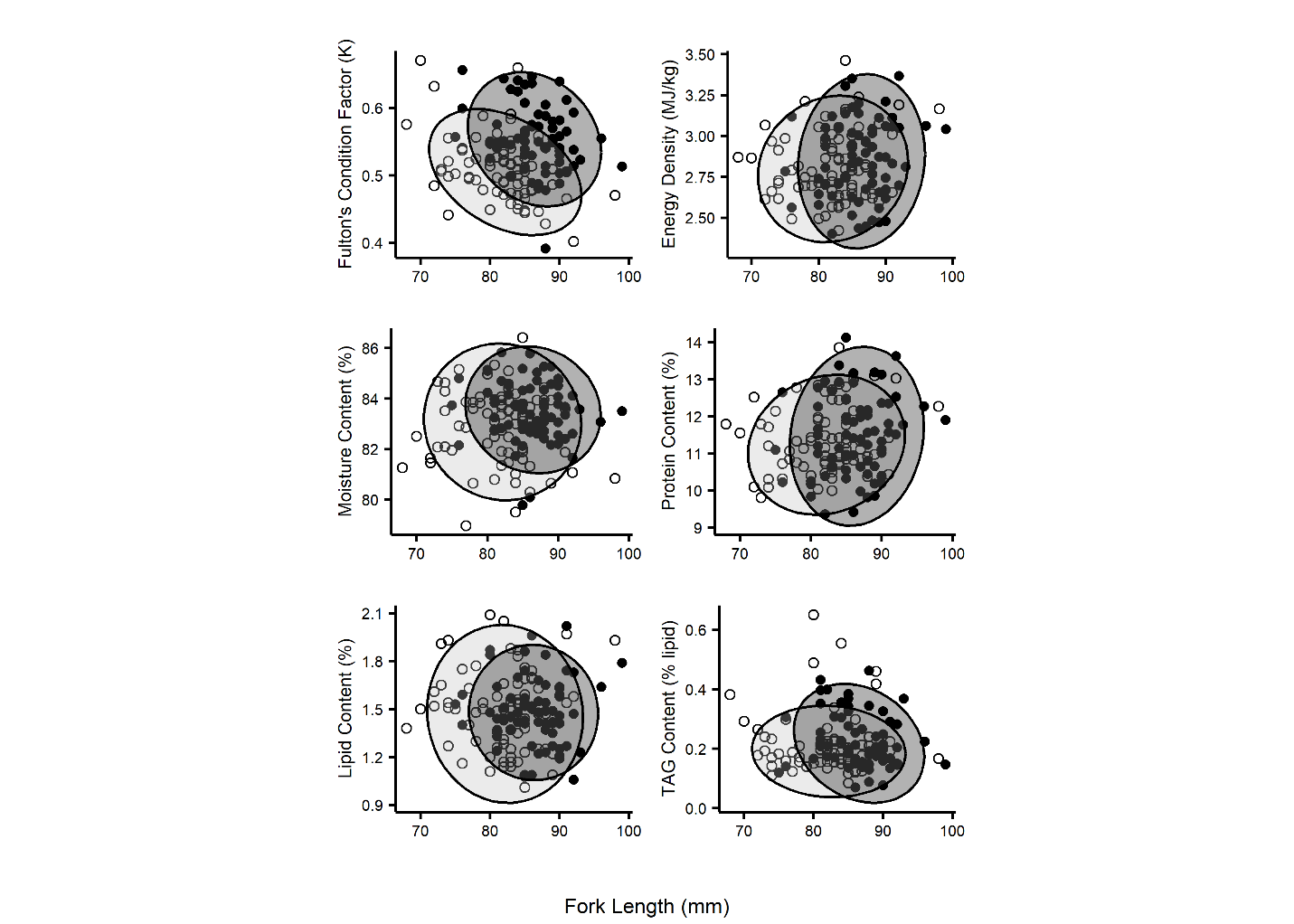 |
| --- |
| Fig S5: Size compared to condition variables for individuals that survived (closed circles) and individuals that died (open circles) during holding period (condition factor, energy density, percent water, percent protein, percent lipid, percent TAG of lipid). Oval region represents 95% confidence region. |

| 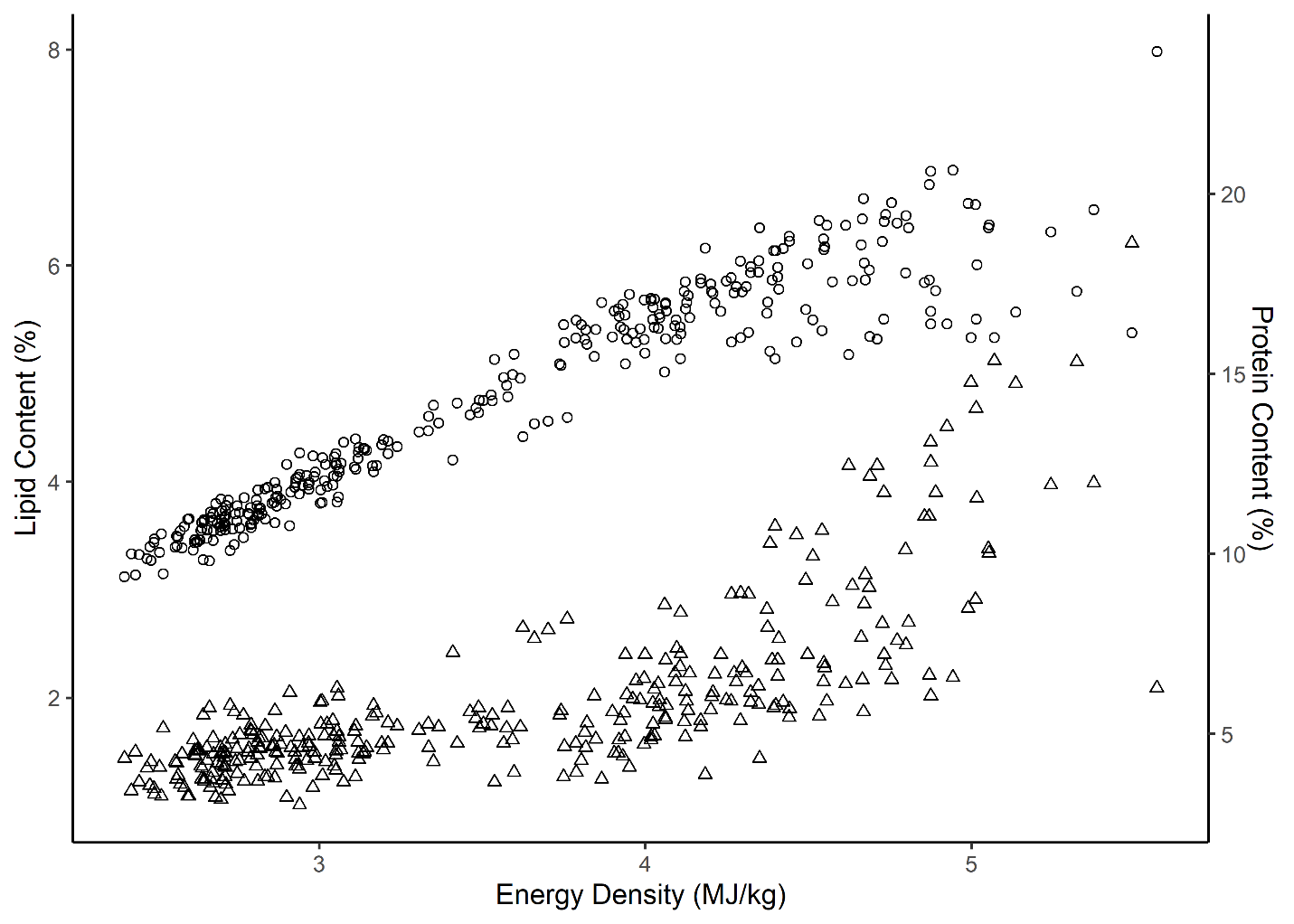 |
| --- |
| Fig. S6: Changes in lipid and protein content contributions to energy density. Triangles represent lipid values, while circles represent protein values. |
